# The Impact of Circadian Preferences on Quality of Life in Patients With Gastroesophageal Reflux Disease: An Evaluation Using the SF‐36

**DOI:** 10.1002/jgh3.70086

**Published:** 2025-01-10

**Authors:** Uğur Ergün, Ahmet Güleç, Taner Buğra Tan

**Affiliations:** ^1^ Department of Internal Medicine Balıkesir Atatürk City Hospital Altıeylül/Balıkesir Türkiye; ^2^ Department of Child and Adolescent Psychiatry Balıkesir Atatürk City Hospital Altıeylül/Balıkesir Türkiye; ^3^ Department of Psychiatry Balıkesir Atatürk City Hospital Altıeylül/Balıkesir Türkiye

**Keywords:** chronotype, circadian preference, gastroesophageal reflux disease, mediation analysis, quality of life, SF‐36

## Abstract

**Background/Aims:**

Gastroesophageal reflux disease (GERD) is a common gastrointestinal disorder that negatively impacts individuals' quality of life.

**Methods:**

This study investigates the effect of circadian preferences on the quality of life in patients with GERD. A total of 152 participants (80 patients diagnosed with GERD and 72 healthy controls) were included in the study. Participants' circadian preferences were assessed using the morningness‐eveningness questionnaire (MEQ), quality of life was evaluated with the SF‐36 scale, and the severity of GERD symptoms was measured using the gastroesophageal reflux disease quality of life scale (GERD‐QOL).

**Results:**

The study's findings indicate that the quality of life and circadian preferences of GERD patients differ significantly from those of the control group. Correlation analyses revealed a positive relationship between SF‐36 and MEQ, but no direct relationship was found between GERD‐QOL and SF‐36 or MEQ. Mediation analyses demonstrated that the effect of GERD‐QOL on SF‐36 is entirely mediated by circadian preferences.

**Conclusions:**

These results highlight the significant impact of circadian preferences on the quality of life in GERD patients, suggesting that individuals with an evening chronotype may have lower quality of life. Our study contributes to the literature as one of the first to suggest that circadian preferences should be considered in the management of GERD.

## 1 Introduction

Gastroesophageal reflux disease (GERD) is typically characterized by the abnormal reflux of stomach contents into the esophagus due to the failure of the normal anti‐reflux barrier. It is one of the most common diseases of the upper gastrointestinal system [1]. GERD often presents as either an erosive or non‐erosive condition, accompanied by symptoms such as dysphagia, odynophagia, pyrosis, cough, hoarseness, and acid regurgitation [2]. The prevalence of GERD in our society is approximately 22.8%, and its widespread occurrence continues [3]. Risk factors predominantly related to patient lifestyle, such as age, male gender, race, use of pain relievers, consumption of certain foods, family history, smoking, obesity, and a sedentary lifestyle, play a significant role in the increasing prevalence of GERD [4].

The progression and symptoms of the disease can significantly impact individuals' daily quality of life [5]. The symptoms experienced by patients, the severity of these symptoms, and any resulting complications can impose various limitations, affecting dietary habits, causing sleep deprivation, and creating problems in family and work life. Consequently, these factors have been reported to negatively influence patients' quality of life [6]. It is essential to assess the quality of life in individuals with GERD and to examine the factors that influence it [7]. Similarly, throughout the course of the disease, there are differences in patients' lifestyles, sleep behaviors, dietary habits, and physical activities, which in turn determine their chronotype preferences [8].

Chronotype is a concept that determines the timing of individuals' daily activities and sleep patterns based on their biological clocks [9]. Chronotypes are categorized into three groups: morning types, evening types, and intermediate types [10]. Morning types are individuals who go to bed early and wake up early, exhibiting peak cognitive and physical performance in the morning, while experiencing decreased performance and fatigue in the evening [11]. Evening types, on the other hand, are those who go to bed late and struggle to wake up in the morning, with their cognitive and physical performance improving in the evening. Intermediate types exhibit characteristics of both morning and evening types, showing balanced performance in both the morning and evening hours [12]. In current literature, two different terminologies are used in this area: chronotype and circadian preference [13]. Chronotype refers to the concept of optimal sleep and activity timing based on individuals' biological clocks, usually measured through objective methods such as actigraphy or melatonin level assessments [14]. In contrast, circadian preference refers to the time periods during which individuals prefer to perform their daily activities, typically assessed through self‐report questionnaires [15]. In our study, the concept of chronotype is addressed as circadian preference, and this term has been used.

Studies examining the relationship between GERD and chronotype primarily investigate the effects of circadian rhythm disruption on GERD [16]. In a multicenter study conducted in Japan by Kiyotoki et al., it was observed that individuals with a late chronotype were more likely to suffer from gastrointestinal disorders such as functional dyspepsia [17]. Similarly, a meta‐analysis by Chen et al. demonstrated that shift work, particularly night shifts, disrupts circadian rhythms and increases the risk of GERD [16]. These findings suggest that chronotype and circadian preferences play a significant role in the development of GERD and highlight the importance of considering individuals' biological clocks in the management of this disease.

In conclusion, this study hypothesizes that circadian preferences significantly affect the quality of life in individuals with GERD. It is proposed that evening‐type individuals may have a lower quality of life compared to morning‐type individuals due to the severity of GERD symptoms. Moreover, it is assumed that circadian preference acts as a mediator in the relationship between GERD and quality of life, as assessed by the SF‐36 quality of life scale. These hypotheses will be tested in alignment with the study's objectives.

## 2 Methods

### 2.1 Subjects and Study Design

The inclusion and exclusion criteria were carefully defined to ensure a well‐matched patient and control group, as well as to enhance the study's internal validity by minimizing confounding factors. Eligible participants were those aged 18 years or older with a confirmed GERD diagnosis based on clinical evaluation, and they had to voluntarily consent to participate. The control group comprised individuals who attended the Internal Medicine Clinic of Balıkesir Atatürk City Hospital without any GERD diagnosis or underlying health conditions. Controls were selected to closely resemble the patient group in terms of demographic characteristics, such as age and gender distribution, allowing for meaningful comparisons.

Exclusion criteria were established to minimize potential influences on the study outcomes. Participants were excluded if they had severe chronic illnesses (such as uncontrolled diabetes, chronic kidney disease, or chronic liver disease) that could independently impact quality of life or circadian preferences. Pregnant or breastfeeding women were excluded due to hormonal and lifestyle factors that could affect the study outcomes. Additionally, individuals with a history of neurological or psychiatric disorders, those on long‐term steroid or immunosuppressive therapy, and participants who had undergone recent gastrointestinal surgery (within the past 3 months) were also excluded to avoid factors that could interfere with circadian rhythms or GERD symptoms. Active smokers and alcohol users were excluded to prevent the effects of these substances on GERD symptoms and circadian patterns. For the control group specifically, individuals were required to have no history of gastrointestinal disease and to be free of related symptoms. These criteria were meticulously implemented to ensure a homogeneous study population and mitigate potential biases that could influence the study's results.

The aim of the study was to examine the impact of circadian preferences on the quality of life in GERD patients. Comprehensive data were collected regarding participants' socio‐demographic information, medical histories, and lifestyles. To collect this data, the Socio‐Demographic Data Form, the SF‐36 Quality of Life Scale, the gastroesophageal reflux disease quality of life scale (GERD‐QOL) for GERD Symptom Severity, and the morningness‐eveningness questionnaire (MEQ) were utilized. Ethical approval for the study was obtained from Balıkesir Atatürk City Hospital, and written informed consent was acquired from all participants.

### 2.2 Socio‐Demographic Data Form

The socio‐demographic data form is designed to collect essential information about the participants, including their age, gender, marital status, educational level, occupation, income level, and smoking and alcohol consumption habits. Additionally, this form gathers information on the participants' general health status, existing chronic diseases, family history of GERD, and lifestyle habits. These data will be used to gain a better understanding of the impact of GERD on individuals' quality of life.

### 2.3 Data Collection Tools

#### 2.3.1 SF‐36 Quality of Life Scale

This scale is a comprehensive tool used to assess the physical and mental health of participants. The SF‐36 consists of eight subscales: physical functioning, social functioning, physical role limitations, emotional role limitations, mental health, energy/vitality, pain, and general health perception. Each subscale enables an objective evaluation of individuals' overall quality of life. It has been observed that this scale is effective in assessing the quality of life in individuals with GERD [18].

#### 2.3.2 Gastroesophageal Reflux Disease Quality of Life Scale

This questionnaire is used to measure the frequency and severity of GERD symptoms, providing a detailed evaluation of how various symptoms impact quality of life. The scale covers common GERD symptoms such as dysphagia (difficulty swallowing), odynophagia (painful swallowing), pyrosis (heartburn), cough, hoarseness, and acid regurgitation. Participants are asked to indicate how often they experience these symptoms and to rate their severity. Additionally, they evaluate the impact of these symptoms on daily activities, social interactions, and overall quality of life. The validity and reliability of the Turkish version of this scale have been established, confirming both its accuracy and reliability [18]. This questionnaire serves as a crucial tool for comprehensively assessing the impact of GERD on individuals and is instrumental in planning treatments and interventions aimed at improving patients' quality of life.

#### 2.3.3 Morningness‐Eveningness Questionnaire

Developed by Horne and Östberg in 1976, this scale classifies individuals according to their biological clocks [19]. The MEQ includes questions that determine the preferred times for sleep and wakefulness, as well as the timing of mental and physical activities, aiming to identify individuals' chronotypes (morning type, evening type, intermediate type). The questionnaire consists of 19 Likert‐type items, with higher scores indicating a greater tendency toward morningness. Scores ranging from 59 to 86 indicate a morning type, 42–58 an intermediate type, and 16–41 an evening type. The MEQ is one of the most widely used scales for examining chronobiological characteristics in both patients and healthy individuals and has been adapted into many languages. In a study conducted by Ağargün and colleagues in 2007, the psychometric properties of the scale were tested, demonstrating high validity and reliability (Cronbach's alpha = 0.81) [20].

### 2.4 Statistical Analysis

The data from the study were analyzed using the IBM SPSS v.22 statistical software package. Descriptive statistics were presented as mean ± standard deviation for continuous variables and as frequency and percentage for categorical variables. Since the data within the groups met the conditions for normal distribution, comparisons were made using the Student's t‐test for continuous data, while categorical data were compared using the chi‐square test. Pearson correlation analysis was conducted to evaluate the correlation between SF‐36, MEQ, and GERD‐QOL scores, including all participants in the analysis. A significance level of *p* < 0.05 was considered statistically significant. The significance of the mediation effect was determined using the bootstrap method. Bootstrap scores were calculated using the SPSS Macro Process program, with 5000 resampling iterations selected. A mediation model analysis was applied using the SPSS HAYES extension, where the MEQ was identified as a mediator in the relationship between SF‐36 and GERD‐QOL.

## 3 Results

Upon examining the age and gender distribution of the 152 individuals who participated in this study, it was found that the mean age in the GERD group (*N* = 80) was 34.93 ± 13.23 years, while in the control group (*N* = 72) it was 35.94 ± 13.83 years. The results of the Student's t‐test indicated that there was no statistically significant difference in mean age between the two groups (*p* = 0.986) (*t* = 0.0031). Regarding gender distribution, the GERD group consisted of 42 females and 38 males, while the control group comprised 37 females and 35 males. Analysis using the chi‐square test revealed no significant difference in gender distribution between the groups (*p* = 0.891) (*x* ^2^ = 0.019). These results indicate that the age and gender variables are homogeneously distributed across both groups, suggesting that these variables are unlikely to influence the study's outcomes (Table 1).

**TABLE 1:** Data on age and gender distribution between the two groups.

Variables	GERD	Control	*F*/*x*2	*p*
	*N*:80	*N*:72		
Age	34.93 ± 13.23	35.94 ± 13.83	0.0031^a^	0.986
Gender				
Female	42	37	0.019^b^	0.891
Male	38	35		

^a^ Student's *t*‐test.

^b^ Chi‐square test.

In this study, significant differences were found between the GERD group (*N* = 80) and the control group (*N* = 72) across various variables. When examining MEQ scores, the mean score for the GERD group was 31.25 ± 10.04, while the mean score for the control group was 56.07 ± 7.84, and this difference was statistically significant (*t* = −19.11, *p* < 0.001). According to the SF‐36 results, the mean score for the GERD group was 72.83 ± 13.22, while the mean score for the control group was 95.35 ± 6.7, with this difference also being statistically significant (*t* = −13.43, *p* < 0.001). In terms of GERD‐QOL scores, the GERD group had a mean score of 13.36 ± 4.65, compared to 45.21 ± 11.57 in the control group, and this difference was found to be statistically significant as well (*t* = −21.8, *p* < 0.001).

When comparing weight data, the mean weight of the GERD group was 73.78 ± 17.81 kg, while the control group's mean weight was 74.11 ± 18.12 kg, with no statistically significant difference between the groups (*t* = −0.11, *p* = 0.909). Similarly, when height data were examined, the mean height in the GERD group was 167.83 ± 7.93 cm, and 167.3 ± 8.2 cm in the control group, with this difference also not being statistically significant (*t* = 0.229, *p* = 0.819). These findings indicate that the quality of life and circadian preferences of patients diagnosed with GERD differ significantly from those of the control group. However, no statistically significant differences were found between the groups in physical measurements such as weight and height (Table 2).

**TABLE 2:** Comparison of different variables between the two groups.

Variables	GERD	Control	*t*	*p*
	*N*:80	*N*:72		
MEQ	31.25 ± 10.04	56.07 ± 7.84	−19.11	**< 0.001**
SF‐36	72.83 ± 13.22	95.35 ± 6.7	−13.43	**< 0.001**
GERD‐QOL	13.36 ± 4.65	45.21 ± 11.57	−21.8	**< 0.001**
Weight (kg)	73.78 ± 17.81	74.11 ± 18.12	−0.11	0.909
Height (cm)	167.83 ± 7.93	167.3 ± 8.2	0.229	0.819

Abbreviations: GERD‐QOL, gastroesophageal reflux disease quality of life scale; MEQ, morningness‐eveningness questionnaire; SF‐36, quality of life scale.

The Pearson correlation analysis revealed a moderate positive and statistically significant correlation between SF‐36 scores and MEQ scores (*r* = 0.579, *p* < 0.001). This finding suggests that circadian preferences are significantly associated with quality of life. In contrast, a very weak negative correlation was found between SF‐36 scores and GERD‐QOL scores, and this relationship was not statistically significant (*r* = −0.055, *p* = 0.625). Similarly, a weak negative correlation was identified between MEQ scores and GERD‐QOL scores, and this relationship was also not statistically significant (*r* = −0.156, *p* = 0.167). These results indicate that circadian preferences are strongly related to quality of life, but the severity of GERD symptoms does not have a significant relationship with either quality of life or circadian preferences (Table 3).

**TABLE 3:** Correlations between variables.

	SF‐36	MEQ	GERD‐QOL
SF‐36	0	*r*: 0.579**	*r*: −0.055
		***p* < 0.001**	*p*: 0.625
MEQ	*r*: 0.579**	0	*r*: −0.156
	***p* < 0.001**		*p*: 0.167
GERD‐QOL	*r*: −0.055	−0.156	0
	*p*: 0.625	0.167	

*Note:* The bold values and the asterisks indicates that circadian preferences are significantly associated with quality of life.

Abbreviations: GERD‐QOL, gastroesophageal reflux disease quality of life scale; MEQ, morningness‐eveningness questionnaire; SF‐36, quality of life scale.

**FIGURE 1:**
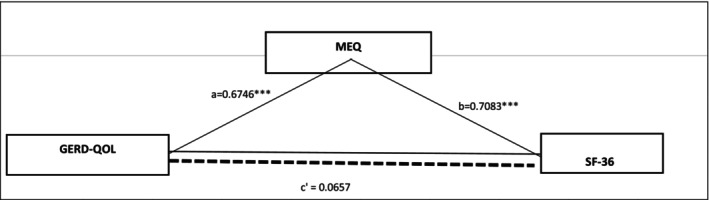
Mediation analysis results. MEQ, morningness‐eveningness questionnaire; GERD‐QOL, gastroesophageal reflux disease quality of life scale; SF‐36: quality of life scale.

In this study, the direct and indirect effects of the GERD‐QOL variable on SF‐36 were examined using Model 4. The analysis was conducted on a sample of 152 participants. Firstly, it was found that the chronotype variable was significantly predicted by GERD‐QOL (*R* = 0.7365, *R* ^2^ = 0.5424, F(1,150) = 177.7884, *p* < 0.0001). The results of the first model indicated that GERD‐QOL had a significant and positive effect on chronotype (*R* = 0.7365, *R* ^2^ = 0.5424, F(1,150) = 177.7884, *p* < 0.001; *β* = 0.6746, *p* < 0.001). In the second model, chronotype was found to have a significant effect on SF‐36 (*R* = 0.8179, *R* ^2^ = 0.6690, F(2,149) = 150.5541, *p* < 0.001; *β* = 0.7083, *p* < 0.001), but the direct effect of GERD‐QOL on SF‐36 was not significant (*β* = 0.0657, *p* = 0.2717). Bootstrap analysis demonstrated that the indirect effect of GERD‐QOL on SF‐36 (mediated by chronotype) was statistically significant (Effect = 0.4779, BootSE = 0.0553, BootLLCI = 0.3790, BootULCI = 0.5928). These findings suggest that the effect of GERD‐QOL on SF‐36 is fully mediated by chronotype, indicating that chronotype plays a complete mediating role (Figure 1).

## 4 Discussion

This study presents significant findings regarding the impact of circadian preferences on the quality of life in individuals with GERD. Our study identified significant differences between the patient and control groups in terms of GERD symptom severity, quality of life, and circadian preferences. Notably, when evaluating the circadian preferences of GERD patients, it was observed that these individuals exhibited a greater tendency toward eveningness. Additionally, the correlation analysis revealed a moderate positive and significant correlation between SF‐36 and MEQ scores, indicating that circadian preferences have a noticeable impact on individuals' quality of life. However, no direct relationship was observed between GERD‐QOL scores and SF‐36 or MEQ. Nonetheless, the mediation analysis determined that the effect of GERD‐QOL on SF‐36 was fully mediated by circadian preferences, suggesting that circadian preference plays a complete mediating role in the impact of GERD on the quality of life of these individuals. Given these findings, our study is the first to demonstrate that circadian preference fully mediates the effect of GERD on the quality of life in individuals with this condition.

GERD is a chronic disease characterized by symptoms and clinical findings associated with mucosal lesion damage caused by the reflux of stomach contents into the esophagus, which reduces the quality of life [21]. The increase in the severity and frequency of disease symptoms negatively affects individuals' standards of living, while the alleviation of clinical findings positively impacts their health‐related well‐being [22]. Today, it is noteworthy that the quality of life of individuals diagnosed with GERD, whose prevalence is increasing day by day, is adversely affected [22, 23]. When examining the impact of patients' circadian preferences, it was determined that evening‐type patients have a lower quality of life compared to morning‐type patients due to the severity of GERD symptoms. Given the impact and duration of GERD symptoms and findings, it is expected that symptom severity would increase in the evening, and such a result is not surprising [24]. To address this, adjusting dietary programs according to appropriate time intervals for patients with reflux may contribute positively to the regulation of reflux symptoms.

It is well‐known that circadian rhythms, whether classified as morning or evening types, affect the quality of life for all individuals, whether healthy or not [25]. A review of scientific literature reveals numerous studies related to circadian rhythms [26]. In our study, we examined the circadian preferences of patients diagnosed with GERD and their impact on quality of life. It was found that the quality of life and circadian preferences of patients with GERD differ significantly from those of healthy individuals. This finding is consistent with the existing literature.

In our study, it was observed that circadian preferences significantly impact individuals' quality of life, with evening‐type individuals being more negatively affected. Similarly, a related study found that individuals with an evening chronotype had lower levels of quality of life [24]. Additionally, in our study, no direct relationship was observed between GERD‐QOL scores and SF‐36 or MEQ. However, mediation analysis revealed that the effect of GERD‐QOL on SF‐36 was entirely mediated by circadian preference, indicating that circadian preference plays a complete mediating role in the impact of GERD on the quality of life in affected individuals. This relationship, identified in the literature, marks our study as the first to highlight that the impact of GERD symptom severity on quality of life is mediated by circadian preference. Various studies have clearly demonstrated that circadian rhythm can influence the severity of GERD symptoms and, consequently, sleep quality [27]. In a related study, it was reported that GERD symptoms are more severe at night due to increased gastric acid secretion and the elevated likelihood of acid reflux in the supine position during sleep [28]. Evening‐type individuals' tendencies to eat or go to bed late can exacerbate these symptoms, leading to a marked decline in sleep quality [29]. Another study noted that sleep disturbances are prevalent among individuals with GERD and that these disturbances negatively affect the overall quality of life [16]. Sleep disturbances in GERD patients are typically associated with an increase in symptoms throughout the night, which can shorten sleep duration or reduce sleep quality, leading to a significant decline in quality of life [30].

Circadian rhythms may significantly influence the symptoms and quality of life in patients with GERD through several potential mechanisms. One prominent mechanism involves the circadian regulation of gastric acid secretion, which typically peaks during the day and declines at night. Evening chronotypes, who experience a misalignment between their biological clock and daily activities, may produce more acid at night when reflux symptoms are more likely to occur, exacerbating their condition. Additionally, circadian rhythms play a critical role in gastrointestinal motility; disruptions in these rhythms can lead to impaired esophageal clearance and delayed gastric emptying, both of which are known contributors to GERD symptom severity. Hormonal fluctuations related to circadian cycles, such as those of cortisol and melatonin, can also affect gastrointestinal function. Increased cortisol levels due to circadian misalignment may exacerbate stress‐related reflux symptoms. Furthermore, lifestyle factors, such as irregular eating patterns and sleep disturbances associated with chronotype preferences, can complicate GERD management, leading to increased symptom frequency and a decline in overall quality of life. Understanding these mechanisms provides a foundation for developing targeted interventions that align treatment strategies with patients' circadian rhythms, ultimately improving their management of GERD [30].

These findings underscore the unique contribution of our study and highlight that circadian rhythms are an important factor to consider in the management of chronic diseases. This awareness, for both healthcare professionals and patients, suggests that lifestyle modifications aligned with circadian preferences could contribute to developing more effective strategies for managing chronic conditions such as GERD.

In conclusion, our findings demonstrate a significant relationship between circadian preferences and quality of life in individuals with GERD, with evening chronotype individuals being particularly negatively impacted. Mediation analyses revealed that the effect of GERD‐QOL scores on SF‐36 is fully mediated by circadian preferences, indicating that circadian preference plays a complete mediating role in the impact of GERD on quality of life. In this context, focusing on lifestyle adjustments in the symptom management of individuals with GERD, particularly changing late‐night eating and sleeping habits, may reduce symptom severity and enhance quality of life. Adjusting dietary and sleep schedules according to appropriate time intervals could also contribute to better symptom control. Increasing healthcare professionals' awareness of the impact of circadian preferences on GERD symptoms could help provide patients with a more holistic and effective treatment approach. In the future, examining circadian preferences in other chronic diseases will be crucial to understanding whether these preferences are a general factor in disease management. Additionally, long‐term monitoring of circadian preferences and quality of life in individuals with GERD will allow us to better understand the trajectory of these relationships over time.

This study has some limitations. First, since the study has a cross‐sectional design, causal relationships between circadian preferences and quality of life cannot be clearly established. This limitation means that the results obtained only demonstrate an association, making it difficult to draw causal inferences. Additionally, the GERD‐QOL used in the study is a self‐report‐based measure. Self‐report scales carry a risk of bias as they rely on participants' perceptions, which may lead to subjective evaluations. Furthermore, the study was conducted in a specific geographic region with a limited sample size, which may restrict the generalizability of the findings and create uncertainty about whether similar results would be obtained in other populations. Moreover, other potential influencing factors that could affect participants' quality of life (e.g., stress levels, diet, physical activity) were not fully controlled. These factors could complicate the relationship between circadian preferences and quality of life, making it difficult to attribute the results solely to circadian preferences. These limitations should be considered when interpreting the findings, and future research should aim to conduct similar studies with broader and more diverse populations using more objective measurement tools.

## Conflicts of Interest

The authors declare no conflicts of interest.
